# Two-Dimensional MX2 Semiconductors for Sub-5 nm Junctionless Field Effect Transistors

**DOI:** 10.3390/ma11030430

**Published:** 2018-03-15

**Authors:** Bin Peng, Wei Zheng, Jiantao Qin, Wanli Zhang

**Affiliations:** State Key Laboratory of Electronic Thin Films and Integrated Devices, University of Electronic Science and Technology of China, Chengdu 610054, China; 201522034014@std.uestc.edu.cn (W.Z.); 201521034028@std.uestc.edu.cn (J.Q.); wlzhang@uestc.edu.cn (W.Z.)

**Keywords:** 2-D materials, monolayer transition metal dichalcogenide FETs, junctionless FETs

## Abstract

Two-dimensional transitional metal dichalcogenide (TMDC) field-effect transistors (FETs) are proposed to be promising for devices scaling beyond silicon-based devices. We explore the different effective mass and bandgap of the channel materials and figure out the possible candidates for high-performance devices with the gate length at 5 nm and below by solving the quantum transport equation self-constantly with the Poisson equation. We find that out of the 14 compounds, MoS_2_, MoSe_2_, and MoTe_2_ may be used in the devices to achieve a good subthreshold swing and a reasonable current ON-OFF ratio and delay. Our work points out the direction of further device optimization for experiments.

## 1. Introduction

With the rapid development of the semiconductor technologies, the transistor will soon go into the sub-10 nm or even sub-5 nm scale in the near future according to the ITRS 2.0 [1,2,3,4]. Unavoidable problems arise, such as heat dissipation and quantum effects, etc. [5,6] The channel materials are crucial to enable high-performance device designs. It was shown that 5-nm will be the limit of channel length in field effect transistors (FETs) based on Si [6]. Among the many possible candidates as suggested in the ITRS [4], 2D materials are the mostly likely to be used in next-generation transistors [7,8] due to their ultimate thickness providing excellent gate control and their dangle bond–free surfaces preventing additional inelastic electron scattering. Their potential excellent properties have been demonstrated by recent simulations [9] and experiments [10,11]. The recent experiment work by Desai et al. demonstrates the possibility of sub-5 nm transistor [11]. However, the performance of such devices depends much on its layout. Two dimensional materials can be used in transistors like FinFET, junctionless FET (JLFET), single or double gated FETs, and so on. he working principle of the so-called junctionless FET was proposed in Canada by Lilienfield [12]. This Lilienfield device is a simple transistor, and the application of the gate voltage modulates its conductivity by depleting the carrier in the channel. It requires that the channel to be thin enough for the gate to completely deplete it. At the same time, a high doping is desirable to carry larger current in the ON-state. It has no junctions that can waive the difficulty to control the doping profile. The junctionless FET [13,14] with its better scalability and fabrication simplicity in comparison to inversion-mode FET [15,16] may be a suitable choice for the future devices. It also shows to have better scalability than an ultra-thin-body (UTB) Si FET due to monolayer its thin body and higher effective mass, resulting in reduced direct source-to-drain tunneling [17]. However, 2D materials must face the main obstacle of controllable doping, and this device should therefore receive more attention. A single layered WSe_2_ p-FET demonstrates a high performance with a sub-threshold slope of 60 mV/dec [18].

Simulations of the device are performed under different approximations, which mainly shows the different ways to handling the Hamiltonian of the channel materials. These methods include full ab initio calculations or the tight binding model with fitting parameters from ab initio calculations and approximation of the low energy part of the electron dispersions with two orbitals. The last approximation requires only two intrinsic parameters to make it work: namely, the bandgap ($E_{g}$) and effective mass of the carrier ($m^{*}$). The crystal configuration of the materials are taken into account at this level. The Hamiltonian of the channel region will give birth to a transmission matrix that can be used to calculate the ballistic transportation of carriers at equilibrium. To simulate the devices characters at a finite voltage, non-equilibrium Green’s function (NEGF) strategies are used in a self-consistent way to solve the electrostatic problems [19]. The NEGF was initiated in the condense matter physics area. With the mean field and single-particle approximation, the many-particle information is casted into self-energies [20].

Quite some works on devices with channel materials of MoS_2_, WS_2_, etc. were shown [21,22]. Their performances were estimated and showed that with channel length about 10 nm and 5 nm they can meet the requirement of ITRS within the next ten years or longer. When the channel is further shortened, the performances degenerate. The gate loses its control due to the tunneling effect which produces too large current at the OFF state of the devices.

Are there better TMCDs that can outperform MoS_2_ and WS_2_? In this work, geometry and material parameters–dependent performances of JLFET were investigated by simulations based on the two band model and the NEGF solvers. Electronic effective masses and the bandgap of the 14 MX_2_ types of transitional metal dichalcogenides were calculated, and the performances of the corresponding materials were mapped. The results show that three MoX_2_ out of the 14 compounds can be used in the future sub-5 nm low-standby-power (LSTP) logical devices.

## 2. Calculation Details

The simulation of the devices is based on the NEGF formalism combined with the Poisson equations, where arbitrary gate geometry and device architecture can be considered. The method is implemented in the open-source code NanoTCAD [23], which is developed by Giuseppe Iannanccone’s group in the University of Pisa. MOSFET with two-dimensional (e.g., graphene, MX2) or three-dimensional channel materials (e.g., CNT) can be simulated. The transportation at finite bias was determined by the Green’s function

(1) $$G\left(E\right)={\left[\left({E+i0}^{+}\right)I-H-\Sigma_{1}-\Sigma_{2}\right]}^{-1}$$

where E is the energy,${i0}^{+}$ is a small number approaching, I is the identity matrix, H is the Hamiltonian for the channel material, and Σ_1,2_ is the self-energy for the source and drain contacts. The self-energies are energy-dependent and can be obtained with a recursive relation for the surface Green’s function [24]. The transmission can be calculated by

(2) $$T\left(E\right)=\mathrm{Trace}\left({G\Gamma }_{1}G^{+}\Gamma_{2}\right)$$

where $\Gamma_{1,2}=i\left(\Sigma_{1,2}-\Sigma_{1,2}^{+}\right)$ is the broadening function of the source and drain. After this, the current is calculated by integration of the current density with respect to the energy.

The electronic structure of the channel materials is modeled with a two-band Hamiltonian ($H_{2D}$). The 2 × 2 matrix is read as

(3) $$H_{2D}=\left[\begin{array}{cc} E_{c} & tf\left(k\right)\\ tf^{*}\left(k\right) & E_{v} \end{array}\right]$$

where $E_{c}$ and $E_{v}$ represent the conduction band minimum(CBM) and the valence band maximum (VBM), respectively. Here *t* represents in-plane hopping energy between two nearest neighboring atoms. The hopping constant t is calculated by $t=\frac{\hbar }{a}\sqrt{\frac{2E_{g}}{3m^{*}}}$. The effective mass ($m^{*}$) was obtained from fitting the dispersions around the CBM to parabolic curves, where the value is calculated by the curvature of the fitting curve with $\frac{1}{m^{*}}=\frac{1}{\hbar^{2}}\cdot \frac{\partial^{2}E\left(k\right)}{\partial k^{2}}$ . The bandgap ($E_{g}$) of the material can be expressed as: $E_{g}=E_{c}-E_{v}$. The structure dependent function f(k) for the hexagonal lattice is written as

(4) $$f\left(k\right)=exp\left(ik_{y}a/\sqrt{3}\right)+2exp\left(-ik_{y}a/2\sqrt{3}\right)\cos\frac{k_{x}a}{2}$$

where $k_{x}$ and $k_{y}$ are the wave vectors in x and y directions of our device, while a denotes the in-plane lattice constant of the compounds. The eigenvalues of the matrix gives the dispersions around CBM and VBM.

(5) $$E^{\pm }\left(k\right)=\frac{\left(E_{c}+E_{v}\right)\pm \sqrt{{\left(E_{c}-E_{v}\right)}^{2}+4t^{2}{\left|f\left(k\right)\right|}^{2}}}{2}$$

In this approximation, the hopping is isotropic. However, some of the MX_2_ compounds show very anisotropic effective mass. Full treatment of the anisotropic bands is beyond the current model. At the same time, the transportation character of the devices in different directions does not vary significantly as shown in the work by Chang [25] where the difference in the mass can be as large as four times. As we can see, the main contributions of the anisotropic effective mass is the density of states where the averaged mass is a constant and can be factored out of the integrand. In the case where we treat the relative variation of the current, the factors cancel. In this case, the effective mass in the transportation direction plays the determined role.

The model input parameters are obtained by separate calculations using the open source code Pwscf [26]. The local density approximation (LDA) functional was chosen to be that parameterized by Perdew and Wang [27]. The plane-wave kinetic energy cutoff was set to 30 Ry with density cut-off of 300 Ry. A shifted 17 × 17 × 2 Monkhorst-Pack mesh was used to perform Brillouin zone integration in order to ensure the convergence of the results. Convergence of the total energy was set to be better than $10^{-8}$ Hartree. Supercells with the vacuum layer thickness of 30 a·u. was used to model the 2D nature of the compounds where periodical boundary of 3D was used [28].

**Figure 1:**
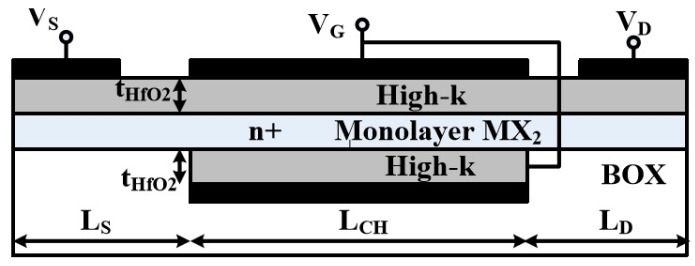
Schematic outlines of the double gated junctionless field-effect transistor (JLFET).

In addition to the channel materials, the oxides play an important role to improve the performances of the devices [29]. Increment of the dielectric constant and reduction of its thickness can reduce the subthreshold swing and increase the gate controllability. In our simulations, the dielectric constant ε is kept at 25 with its thickness of 2.0 nm without further optimization. The ε is the value of high-κ material like HfO_2_. The schematics of JLFET in the double-gate (DG) configuration with a monolayer MX_2_ channel are shown in Figure 1. The lengths of source and drain are 5 nm. JLFET consists of a uniformly doped source, drain, and channel with a molar fraction equal to $10^{-2}$ corresponding to the doping level about $3\times 10^{13}\;{\mathrm{cm}}^{-2}$. In the model, the metallic contacts to the source and drain are overlooked. It may be included in a device model as shown by Agarwal et al. [3]. The contact resistance will mainly decrease the ON-state current. It may reduce the current by one order when the resistance increases from 10 to 1000 Ω·μm. This will thus increase the intrinsic delay of the devices.

## 3. Subthreshold Swing

As one of the most important figure of merits (FOM), the subthreshold swing (SS) of the devices is defined as the gate voltage ($V_{GS}$) swing needed to change the drain current Id by one decade. Hence, we calculated the *SS* numerically by

(6) $$SS\equiv \frac{dV_{GS}}{dlgI_{d}}$$

**Figure 2:**
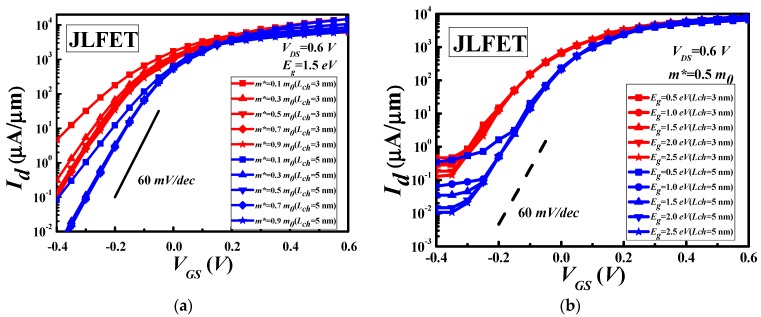
The transfer characteristics of JLFET with different lengths of channel and different bandgap for fixed $E_{g}$ (**a**) and $m^{*}$ (**b**), respectively.

The source to drain current $I_{d}$ at different gate voltages $V_{GS}$ with bandgap, effective mass, and channel length as parameters are shown in Figure 2. It is clearly observed that the shorter channel length produces too high leakage current at the order of 0.1 μA/μm, while the saturation current is at the same order of 10^4^ μA/μm. This value is determined by the available modes of electrons in the ballistic transportation, which is roughly proportional to the DOS of the channel. According to our simulation shown in the figure, for devices with the channel length down to 1.0 nm, it is impossible to achieve good performances in our parameter range. The current at the OFF state is too large due to the tunneling effect with such a short channel length. This tunneling effect manifests it by the differences in Figure 2a,b. As can been seen in Figure 2a, the effective mass can influence the current widely when the bandgap is fixed to 1.5 eV. On the other hand, when the bandgap changed from 0.5 eV to 2.5 eV, the source to drain current $I_{d}$ at the same gate voltage changes very subtle when the effective mass is fixed at 0.5 $m_{0}$ as shown in Figure 2b. The curves clustered with respect to the channel length, which is obvious in the figure.

**Figure 3:**
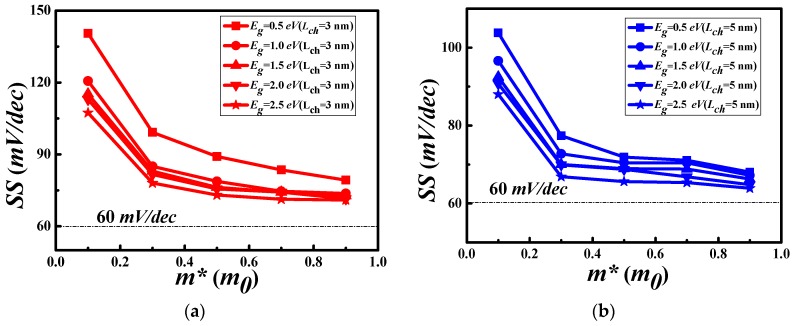
SS vs. the electron effective masses with different bandgap ($E_{g}$ ) for channel length ($L_{ch}$ ) of 3 nm (**a**) and 5 nm (**b**), respectively.

The SS versus the electron effective mass ($m^{*}$) and the bandgap at channel length of 3 nm and 5 nm are shown in Figure 3. The thermionic limit of 60 mV/dec is shown by the dot-dashed horizontal line. Both devices approach to the thermonic limit when the effective mass is increased, which means that the controllability of the gate is better with the larger carrier effective mass. It is natural to achieve lower SS when the effective mass is larger. This is because the tunneling current is the main source to worsen the SS. It is reduced when the effective mass is increased because of the reduction of the current.

## 4. Other FOMs of the Devices

**Figure 4:**
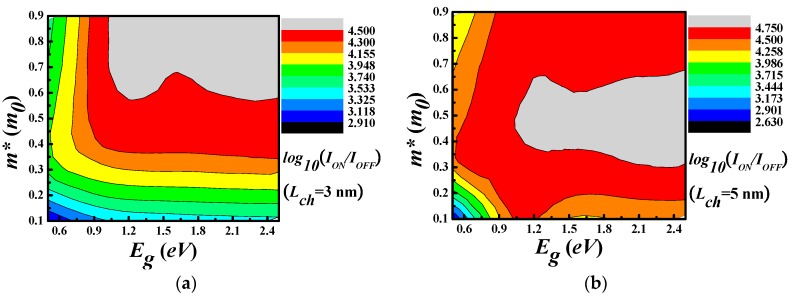
The $I_{ON}/I_{OFF}$ of devices with different channel length (**a**) $L_{ch}=3\;\mathrm{nm}$ and (**b**) $L_{ch}=5\;\mathrm{nm}$ as a function of bandgap and effective mass.

To further estimate the FOMs of 2D JLFET, we compute the ratio $I_{ON}/I_{OFF}$ where $I_{OFF}$ denotes the $I_{d}$ in the OFF state for the n-type FETs ($V_{GS}^{OFF}=V_{GS|I_{OFF}=0.1\mu A/\mu m},V_{GS}=V_{DD}$ where $V_{DD}$ is the supply voltage) and $I_{ON}$ is the current in the ON state ($V_{GS}^{ON}=V_{DD}+V_{GS}^{OFF},V_{GS}=V_{DD}$). According to the ITRS this ratio has to be larger than 10^4^ to ensure a good balance between the static and dynamic power consumption. The ON-OFF ratio with different $m^{*}$ and bandgap $E_{g}$ were shown in Figure 4a,b. The “L”-shape contour line with its leg on the bandgap side means that the ratio is insensitive the bandgap when it is about 1.0 eV for both channel length. However, when it is smaller than the 1.0 eV, it is insensitive to the effective mass. The large $I_{ON}/I_{OFF}$ can be achieved when the effective mass is between 0.6 and 0.8 $m_{0}$, while this value is reduced to 0.4–0.6 when the channel length is increased from 3 nm to 5 nm. When compared with the prediction of ITRS for 2026 LOP devices, the requirement of the ratio is twice as the highest achievable ratio within our parameter space. It is at the same order in UTB Si devices as shown by Alam et al. [21]. As shown by recent work [11], this effective channel can be down to 1.0 nm, while the ON-OFF ratio can be larger than 10^4^.

The speed and the energy consumption are also important parameters for HP logic devices. The intrinsic delay is a measurement of switching speed, which is calculated as

(7) $$\tau =\frac{Q_{ON}-Q_{OFF}}{I_{ON}}$$

where $Q_{ON}$ and $Q_{OFF}$ are the mobile charge Q in the whole device at the ON and OFF state, respectively. Considering the energy consumption, we calculate the Power-Delay Product (PDP) as

(8) $$PDP=V_{DD}I_{ON}\tau =V_{DD}(Q_{ON}-Q_{OFF})$$

**Figure 5:**
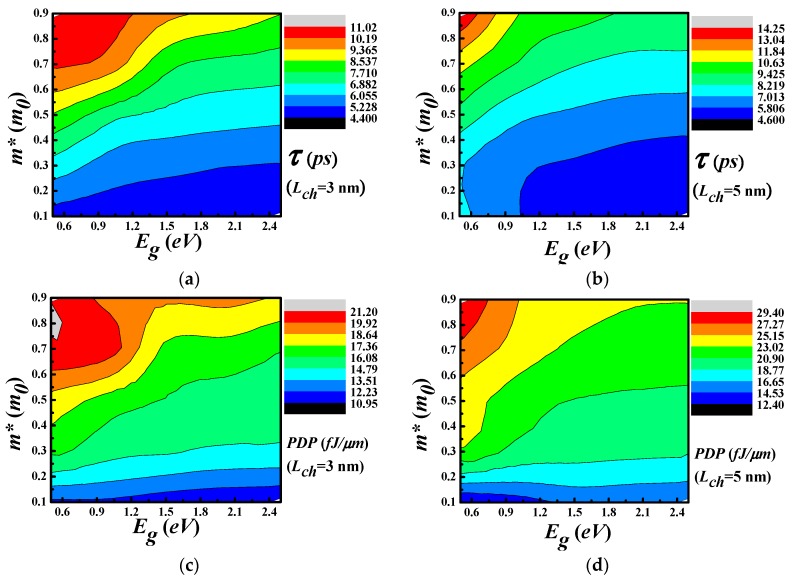
The delay (**a**,**b**) and PDP (**c**,**d**) of the devices as a function of the bandgap at different channel length of 3 nm and 5 nm.

The PDP denotes the switching energy used in logic devices. During this calculation, the $I_{OFF}$ is set to 0.1 μA/μm, which is the same as the ITRS requirement for the HP applications. Both the τ and PDP of the different devices with channel length of 3 nm and 5 nm are functions of the bandgap and effective masses, as shown in Figure 5. The minimum time delay achievable is about 5.0 ps with respect to most of the bandgap ($E_{g}$). It is a monotonic decrease function of the effective mass as shown in (a) and (b) in the Figure 5. This gives us the flexibility to choose suitable materials. However, the delay is several times larger than the requirement of the HP devices in ITRS2.0. Reduction of the delay requires larger ON-state current, as shown in the above equation, so that the charging process can be faster. Increasing the current can be realized by a higher doping level or using a multilayer of 2D materials.

The decrease of the delay with respect to the effective mass points to the conclusion that the gate charge variation from the OFF-state to the ON-state is much reduced when the carriers are heavier. The contour of PDP shows that it is mainly determined by the effective mass and a smaller $m^{*}$ is preferred in order to decease the PDP. It is obvious that the two FOMs have a contradictory dependence on the effective mass while a fare tolerance of the bandgap. The shorter channel length will benefit the power efficiency as expected when we compare the data from the devices with the two channel length. However, the reduction is only marginal.

## 5. Proposal of the Materials

**Figure 6:**
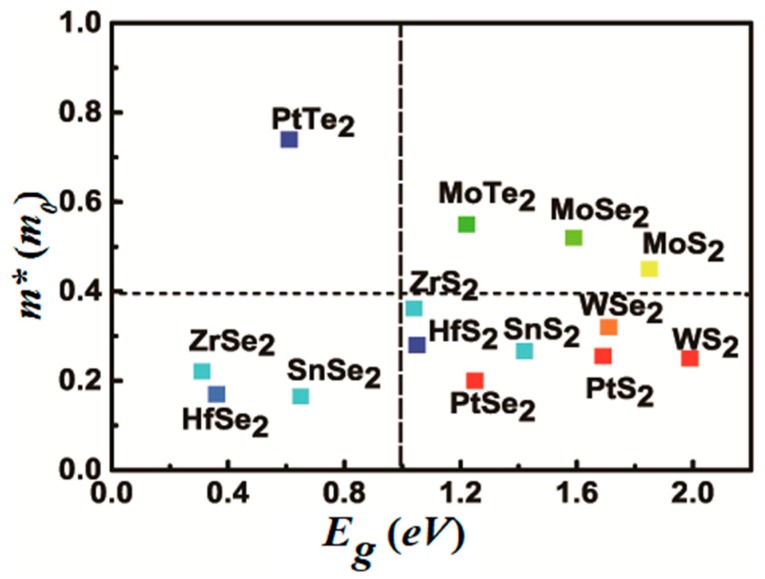
The chart of the electron effective masses and their bandgap of the 14 MX_2_ single layer semiconductors.

With respect to these results, we next figure out the possible material candidates for channel materials in logic FETs, MX_2_ covered a wide range of bandgap and effective carrier masses. We plot a chart of the electron effective masses versus the bandgap predicted by DFT [30] in Figure 6. For the anisotropic materials, we use the smaller effective mass, since it determines the SS for the current transportation in this direction as argued above. The horizontal and vertical dashed line in Figure 6 denote the effective electron mass of 0.4 $m_{0}$ and bandgap 1.0. With the $m^{*}$ above 0.4 $m_{0}$, the SS can be reduced to about 70 mV/dec for a 5nm channel length and 80 mV/dec for 3 nm., the ON-OFF ratio can reach 5 × 10^4^ when the bandgap is above 0.9 eV even when the channel length $L_{ch}$ is only 3 nm. With these material parameters, the delay can be reduced to 7 ps and the PDP is below 18 fJ/μm. From the consideration above, we can see only three compounds locate at the upper-right corner of the chart. They are all molybdenum dicholgenides compounds. These compounds are promising when used in the devices with channel length below 5 nm. For the others, it is difficult to balance the different devices performances. Although PtSe_2_ and HfSe_2_ have ultra-small effective masses [31] that were promising for devices working in the long channel, they are not ideal to work in the ballistic transport regions. As the SS in JLFET is limited by the thermionic emission of the electrons in the source, it is impossible for JLFET to go beyond that. In order to suppress the thermionic limitation of the devices, a new concept is needed, such as the band-to-band tunneling mechanism in 2D materials [5,31] or control of the source and channel region separately, as shown very recently [32].

## 6. Conclusions

Combining the ab initio calculations and the NEGF equations, we calculated the performances of JLFET in order to figure out which 2D materials are suitable for next-generation logic devices with a channel length smaller than 5 nm. Two main material parameters—the bandgap and the effective mass—are taken into account. We find that MoX_2_ (X = S, Se, and Te) may be used in the devices to achieve reasonable performances. However, the intrinsic delay and power-delay products are one order of magnitude larger than the goal of the ITRS2.0. A novel device concept and design should be used to make full use of the merits of 2D material in logical devices.
